# Retina Is Protected by Neuroserpin from Ischemic/Reperfusion-Induced Injury Independent of Tissue-Type Plasminogen Activator

**DOI:** 10.1371/journal.pone.0130440

**Published:** 2015-07-15

**Authors:** R. P. Gu, L. L. Fu, C. H. Jiang, Y. F. Xu, X. Wang, J. Yu

**Affiliations:** 1 Department of Ophthalmology and Vision Sciences and Key Laboratory of Myopia of State Health Ministry, Eye and ENT Hospital, Shanghai Medical College, Fudan University, Shanghai, 200031, China; 2 Department of Ophthalmology, Union Hospital, Tongji Medical College, Huazhong University of Science and Technology, Wuhan, China; 3 Department of Ophthalmology, No. 5 people’s Hospital of Shanghai, Shanghai, 200240, China; Universidade Federal do Rio de Janeiro, BRAZIL

## Abstract

The purpose of the present study was to investigate the potential neuroprotective effect of neuroserpin (NSP) on acute retinal ischemic/reperfusion-induced (IR) injury. An IR injury model was established by elevating intraocular pressure (IOP) for 60 minutes in wild type and tPA-deficient (tPA-/-) mice. Prior to IR injury, 1 μL of 20 μmol/L NSP or an equal volume of bovine serum albumin (BSA) was intravitreally administered. Retinal function was evaluated by electroretinograph (ERG) and the number of apoptotic neurons was determined via TUNEL labeling. Caspase-3, -8, -9,poly (ADP-ribose) polymerase (PARP)and their cleaved forms were subsequently analyzed. It was found that IR injury significantly damaged retinal function, inducing apoptosis in the retina, while NSP attenuated the loss of retinal function and significantly reduced the number of apoptotic neurons in both wild type and tPA-/- mice. The levels of cleaved caspase-3, cleaved PARP (the substrate of caspase-3) and caspase-9 (the modulator of the caspase-3), which had increased following IR injury, were significantly inhibited by NSP in both wild type and tPA^-/-^ mice. NSP increased ischemic tolerance in the retina at least partially by inhibiting the intrinsic cell death signaling pathway of caspase-3. It was therefore concluded that the protective effect of neuroserpin maybe independent from its canonical interaction with a tissue-type plasminogen activator.

## Introduction

A member of the serine family, tissue-type plasminogen activator (tPA) is responsible for the conversion of plasminogen to plasmin, an enzyme that plays a role in the degradation of proteins in plasma. Thus, tPA can be critical for the treatment of vascular diseases ofthe central nervous system[1,2,3],as well as the visual system[4,5,6]. Interestingly, studies have demonstrated that tPA may also contribute to neural toxicity by increasing vascular permeability, inflammation reactions and by inducing excitotxic cell death[7,8,9,10]. As such, there has been much interest in research toward the finding of agents which can combat such toxicity. tPA has many natural antagonists. One of these is neuroserpin (NSP)[11,12]. A serine proteinase inhibitor, NSP has been reported to react preferentially with tPA, and is primarily expressed by the neurons in the central nervous system[11,12,13]. It has also been previously reported that NSP plays a role in the regulation of neuronal growth and maturation[12,13,14].In addition, NSP has demonstrated a neuroprotective effect during stroke and other pathological conditions of the central nervous system[15,16,17]. It remains unclear, however, whether NSP has such neuroprotective effects on pressure-induced ischemic-reperfusion (IR) injury in the retina, which has been generally accepted as one of models for central retinal artery occlusion and acute angle closure glaucoma due to similarities in pathology[18,19].

The retinal IR injury of a mouse can be established by elevating intraocular pressure through cannulation of the eye to interfere with retinal circulation. This is followed by a natural reperfusion response[18,19].Electroretinography (ERG) has demonstrated decreased a-wave and b-wave activity after this type of ischemic event, with the b-wave predominantly affected[20]. Histologically, the IR model has been shown to induce apoptosis in retinal neurons and a decreased thickness of retinal cell layers, especially the inner retinal layer[21]. In the present study, it was found that NSP protects the retina from ischemic injury as measured by functional and morphometric analysis. Further, NSP’s neuroprotective effect on the retina may be rendered by the inhibition of the caspase-9 signaling pathway, a pathway that has been implicated in regulating cell death. This effect appears to act independently of tPA inhibition.

## Materials and Methods

### Ethics statement

This study was carried out in strict accordance with the Guidelines on the Care and Use of Laboratory Animals issued by the Chinese Council on Animal Research and the Guidelines of Animal Care. The protocol was approved by the Committee on the Ethics of Animal Experiments of Fudan University. All mice were anesthetized intraperitoneally with xylazine (20 mg/kg) and ketamine (80 mg/kg) before all surgical procedures. All efforts were made to minimize the animals’ suffering and to reduce the number of animals used.

### Animal Use

All procedures for this study were approved by the Animal Ethics Committee of the Eye and ENT Hospital of Fudan University, China, and conducted in accordance with the Association for Research in Vision and Ophthalmology (ARVO)’s statement on the use of animals in ophthalmic and vision research. A total number of98male mice were used: sixty two wild type (C57BL/6) and thirty six tPA-deficient mice (tPA-/-; provided by Prof. Song HY, Department of Molecular Genetics, Fudan University, China)[22](S1 Fig).Mice were aged between 8–12 weeks and weighed16–20g each. The mice were raised under normal 12-hour cyclic illumination and with free access to water and standard mouse chow.

Twelve wild type mice were randomly chosen and equally divided into the IR+NSP or IR+BSA control group for ERG. Another12 wild type mice were randomly and equally divided into four groups for TUNEL staining: untreated, IR, IR+BSA and IR+NSP. Twenty more mice were randomly divided into the same four groups for apoptotic signal pathway analysis via western blot. Finally, 9 wild type mice were randomly assigned for NSP expression analysis via immunofluorescence (n = 3/time point of 24 hours) and 9 for NSP expression utilizing western blot (n = 3/time point of 24 hours).A total number of 36tPA-/- mice were used. Twelve were randomly and equally divided into IR+NSP and IR+BSA groups for ERG, 9 equally divided into three groups (untreated, IR+BSA and IR+NSP)for TUNEL staining, and 15 divided into the same three groups for western blot analysis of apoptotic indicators(S1 Table).

### Intravitreal injection

Though endogenous NSP may be produced 0–24 hours after IR injury, these may not be sufficient for neuroprotection (S2 Fig). Seemingly, optimal concentrations for neural protection after hypoxia-induced injury occur at0.8–1umol/L[23]. Considering that the volume of the mouse vitreous is 20ul, an intravitreal injection of 1ul NSP(20umol/L) was chosen to achieve an NSP concentration of 1umol/L. Specifically, IR+NSP groups received an intravitreal injection of 1μL NSP solution (20μmol/L)[24,25],while IR+BSA control groups received an intravitreal injection of 1μL 0.1% BSA. NSP solution (20μmol/L) was prepared by dissolving human recombinant NSP (PeproTech, USA) in 0.1%BSA.A punch incision was made0.5mm posterior to the limbus using a 30-gauge needle through which a micro injector was inserted pointing toward the optic papilla. When it was confirmed that the needle tip had reached the vitreous, an intravitreal injection of NSP was delivered. Subsequently, anIR injury protocol was immediately performed.

### IR injury in Retina

IR injury was induced in IR+BSA and IR+NSP groups upon intravitreal injection of either NSP or BSA.Non pre-treated IR groups were subject to IR injury directly. A transient retinal IR injury was induced in the right eye, with the left eye serving as an un-injured control(described previously)[26]. All mice were anesthetized and the pupils of the right eyes were dilated. The anterior chambers were canulated with a 33-gauge infusion needle which was connected to a physiological saline bottle. When the saline bottle was elevated, the intraocular pressure (IOP) was raised to 120 mmHg. The retinas were monitored for evidence of blanching, which indicated a loss of blood flow. After 60-min ischemia, the needle was withdrawn before reperfusion.

### Electroretinogram (ERG)

ERG measurements were conducted at baseline and after IR injury at 24 hours and 7 days post-injury. IR+NSP and IR+BSA groups(wild type n = 12; tPA-/- n = 12) were adapted to the dark overnight, before intraperitoneal anesthesia with xylazine (20 mg/kg) and ketamine (80 mg/kg). The pupils were dilated with phenylephrine hydrochloride (2.5%) and atropine sulfate (1%) followed by the placement of two contact lens electrodes onto both eyes so that full-field ERGs could be recorded. Recordings were made using the Espion Visual Electrophysiology System (Espion E^3^, Diagnosys, Diagnosys UK Ltd, UK). Single flashes (10 ms) with an intensity of 2.5 cd-s/m^2^ were used for retinal stimulation under the scotopic condition.

### TUNEL staining methods

The number of apoptotic neurons in un-treated controls, IR, IR+BSA and IR+NSP groups was counted 24 hours after IR injury via TUNEL staining. The right eyes were enucleated, and with the removal of the cornea and lenses, the eyecups were immersed in 4% paraformaldehyde (PFA) for 2 hours. Next, the eyecups were dehydrated in graded sucrose solutions (20%-30%) and embedded in OCT compounds (Tissue-Tek; Ted Pella Inc, Redding, CA, USA).The eye cups were snap-frozen at -80°C and 20 minutes later, were sectioned (10μm) before air-drying at room temperature.

Sections were then treated with reagents from the In Situ Cell Death Detection Kit, Fluorescein (Roche, USA). Briefly, sections were thrice washed in 0.01 M PBS for 10 minutes followed by a 60-minute incubation with 0.1% triton X-100. Sections were immersed in TUNEL reaction mixture(50 μL enzyme solution added to 450 μL label solution) at 37°C for 60 minutes. Another PBS was sequence was performed before treatment with a fluorescent medium (DakoCytomation, Denmark).Treated sections were examined under a confocal laser microscope (TCS SP2; Leica Microsystems, Bensheim, Germany) with equilibrated parameters. In each section, two adjacent areas of 0.700mmlengths were selected for imaging, inferior and superior to the optic nerve head. In each area, the number of TUNEL-positive cells was counted and averaged. Only one section was chosen from each eye, and each group contained three eyes.

### Western blots

Western blot was conducted on tissues from 35 mice (20 wild type and 15 tPA-/-) for apoptotic signal pathway analysis and on tissues from 9 wild type animals for NSP expression. The former were euthanized24 hours after IR injury and the latter were euthanized either at baseline (before IR injury), 0hours, or 24hours after IR injury. Upon the removal of the anterior segments, all retinas were harvested and frozen at -80°C. Tissues were mixed with RIPA buffer (Beyotime, China) and ultrasonically smashed to achieve homogenized retinas; the homogenate was incubated on ice for 15 minutes and centrifuged at 12,000× g for 15 minutes at 4°C. The supernatant was collected and the protein concentration was measured with a BCA protein assay (Beyotime, China). Next, the treated supernatant was re-suspended in 5× SDS-PAGE sample buffer (Beyotime) and boiled for 5 minutes.50μg proteins were loaded into each lane, which was separated by 12% sodium dodecyl sulfate polyacrylamide gel electrophoresis and transferred onto a nitrocellulose membrane. Membranes were pre-blocked in 5% non-fat milk at room temperature for one hour and incubated with either a rabbit polyclonal antibody raised against mouse NSP (1:1,000, Abcam), rabbit anti- caspase-3 (1:1,000, Cell Signaling Technology, USA),rabbit anti-cleaved caspase-3 (1:1000, Cell Signaling Technology, USA), rabbit anti- caspase-8 (1:1,000 CST, USA), rabbit anti-cleaved caspase-8 (1:1,000 CST, USA), rabbit anti- caspase-9 (1:1,000 CST, USA), rabbit anti-cleaved caspase-9 (1:1,000 CST, USA), rabbit anti- PARP (1:1,000 CST, USA), rabbit anti-cleaved PARP (1:1,000 CST, USA) or a monoclonal mouse anti-beta-actin antibody (1:1,000, Abcam, Hong Kong) in 1% BSA at 4°C overnight.

Following an incubation of peroxidase-conjugated donkey anti-rabbit secondary antibody (1:20,000Amersham Pharmacia Biotech Ltd., UK), the membranes were treated withch emiluminescence (Pierce, Pierce Biotechnology, Rockford, IL, USA). Chemiluminescent images were captured using a Kodak Image Station 4000MM PRO (Carestream, Rochester, NY, USA)and analyzed with Image-Pro Plus (ver. 6.0, Media Cybernetics, Bethesda, MD, USA).

### Immunofluorescence for NSP

Upon euthanasia, 9 wild type mice were transcardially perfused with saline and4% paraformaldehyde (PFA) at either baseline,0 hours, or 24 hours after IR injury. Previously described methods were used to harvest and section the tissue. Dry sections were immersed in PBS containing 5% goat serum and 0.25% Triton X-100 for 15 minutes then washed thrice with 0.01 M PBS. Sections were next incubated overnight (4°C)with a rabbit polyclonal antibody raised against mouse NSP (1:800 dilution; Abcam, Hong Kong).

After incubation, the sections were washed in 0.01 M PBS and immersed in Alexa Fluor 488-conjugated goat IgG secondary antibody (1:500; Invitrogen, Hong Kong) for one hour at room temperature. After a final wash, sections were treated with a fluorescent medium (DakoCytomation, Denmark)and analyzed under a confocal laser microscope (TCS SP2; Leica Microsystems, Bensheim, Germany) withe quilibrated parameters.

### Data analysis

Statistical analyses were performed. One-way ANOVA analysis was used to compare the number of apoptosis and the relative expression of protein among normal, IR, IR+BSA and IR+NSP groups. Then two-tailed unpaired Student’s *t*-test was performed to further compare the difference between each two groups. And the amplitudes of ERG were compared between two groups treated with NSP and BSA respectively by using two-tailed unpaired Student’s *t*-test. All the data represented in the results were obtained from three or more independent experiments and denote averages and standard deviations (mean ± SD). The value of *p*<0.05 was considered the threshold for statistical significance.

## Results

### The retina is protected by NSP from IR injury in wild type mice

**Fig 1:**
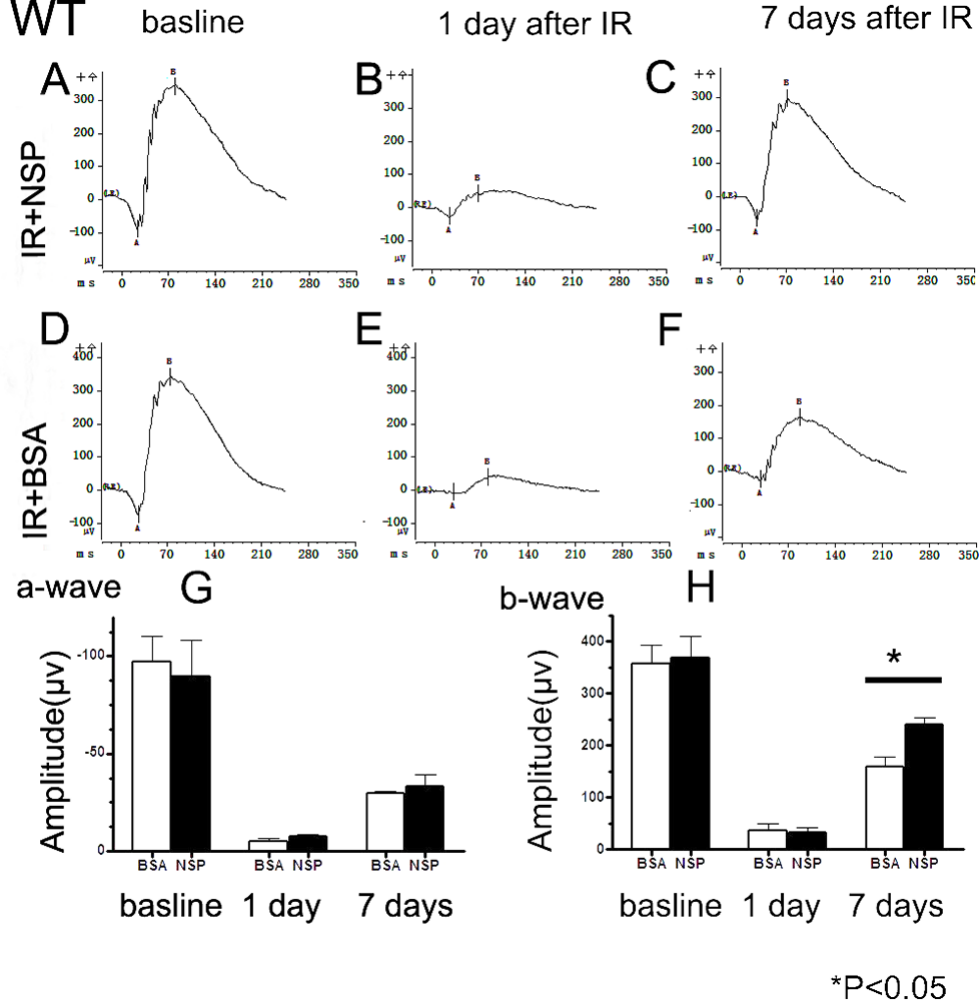
Effect of NSP on ERG responses in wild type mice. (A-C) Representative ERG of wild type mice of the treatment assignment IR+NSP at baseline (before IR injury), 1 day and 7 days after injury, respectively. (D-F) Representative ERG of wild type mice of the treatment assignment IR+BSA at baseline and 1 day and 7 days after ischemic retinal injury. Each ERG was obtained by averaging two responses to 2.5cd.s/m^2^ flashes with an interstimulus interval of 10 milliseconds; (G) Data analyses of ERG a-wave amplitudes for both treatment conditions at baseline and one day and seven days after retinal ischemic injury; (H) Data analyses of ERG b-wave amplitudes at baseline and one day and seven days after retinal ischemic injury.Data is expressed as a mean ±SE; n = 6; (*) *P*<0.05between responses in IR+BSA control and IR+NSP groups.

Scotopic ERG measurements performed on NSP-treated groups and BSA-treated controls revealed similar a- and b-waves at baseline. Twenty-four hours after IR injury, a- and b-waves were significantly attenuated in both animal treatment groups, with no significant differences between the two groups. Seven days post-injury, however, as some recovery was observed in the two groups, the amplitudes of the b-wave became significantly higher for the NSP-treated animals than the BSA-treated controls (*p* = 0.02) (Fig 1, S2 Table).

### IR-induced apoptosis is prevented by NSP in wild type mice

**Fig 2:**
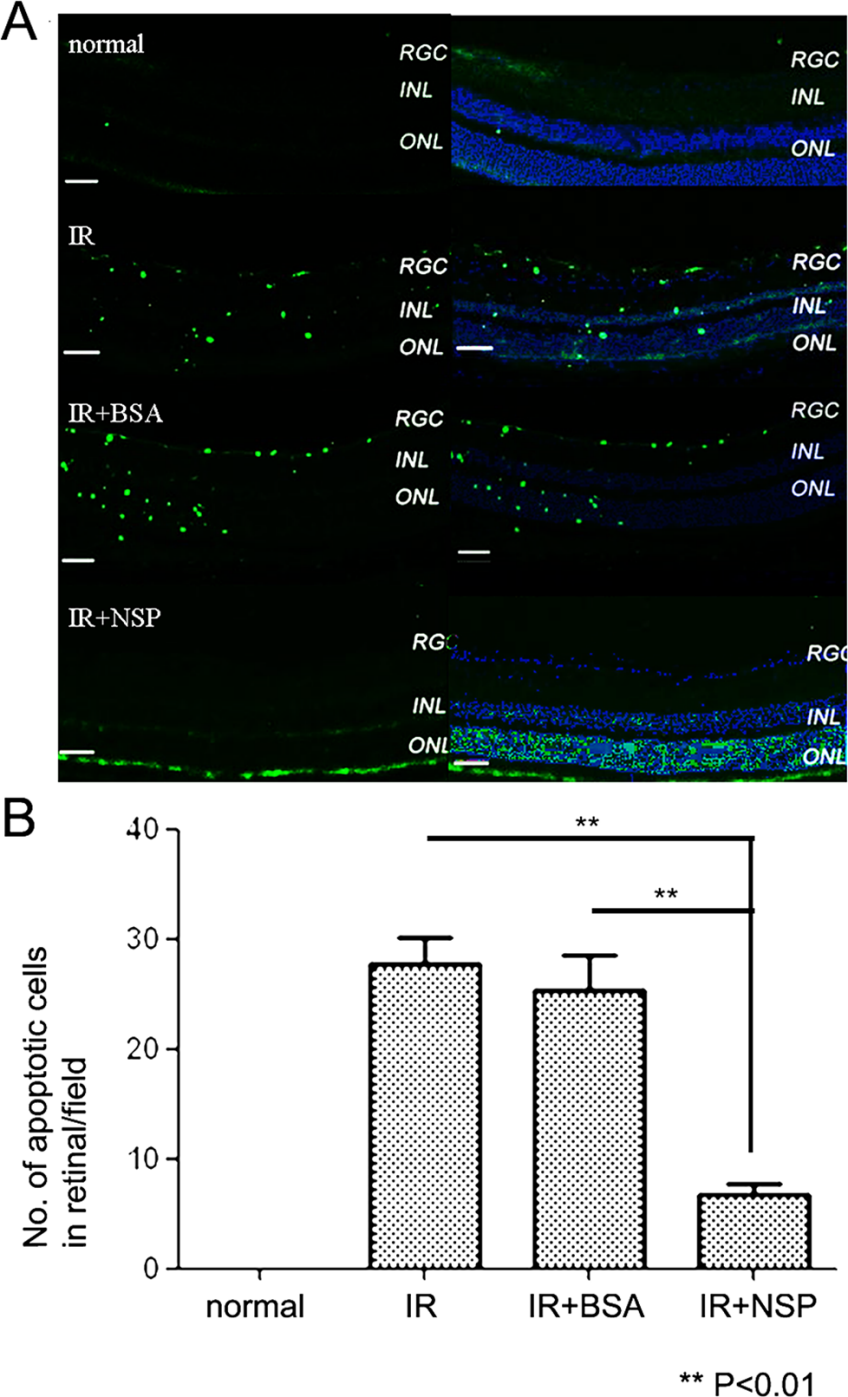
Apoptosisreduction in retina24 hours after IR injury in wild type mice. (A) TUNEL analysis of retinas from four different treatment groups: normal control (top), IR control (upper middle), IR+BSA control (lower middle) or IR+NSP group (bottom). Green indicates TUNEL-positive cells; blue indicates DAPI; RGC: retinal ganglion cell layer, INL: inner nuclear layer, ONL: outer nuclear layer. Scale bar, 50μm(B)Quantitative analysis of TUNEL-positive cells in retinas from different treatment groups. Data are presented as mean and error bars represent standard deviations (SD); Each group n = 3; Asterisks denote significant differences from the TUNEL levels of the untreated IR injury group and the BSA-treated injury control group; (**) *P*<0.01; NSP: neuroserpin, BSA: bovine serum albumin.

Twenty-four hours after IR injury, TUNEL-positive nuclei were observed at different layers of the retina, including the ganglion cell layer (GCL), the inner nuclear layer (INL) and the outer nuclear layer (ONL). The pretreatment of 1 μL NSP solution (20 μmol/L) before IR injury significantly decreased the number of TUNEL-positive nuclei, a decrease which was not achieved in the groups pretreated with BSA (IR+NSP: 6.67±1.15 per field and IR+BSA: 25.33±3.21 per field; *p*<0.01; original magnification 100x) (Fig 2).

### The retina is protected by NSP from IR injury in tPA^-/-^ mice

**Fig 3:**
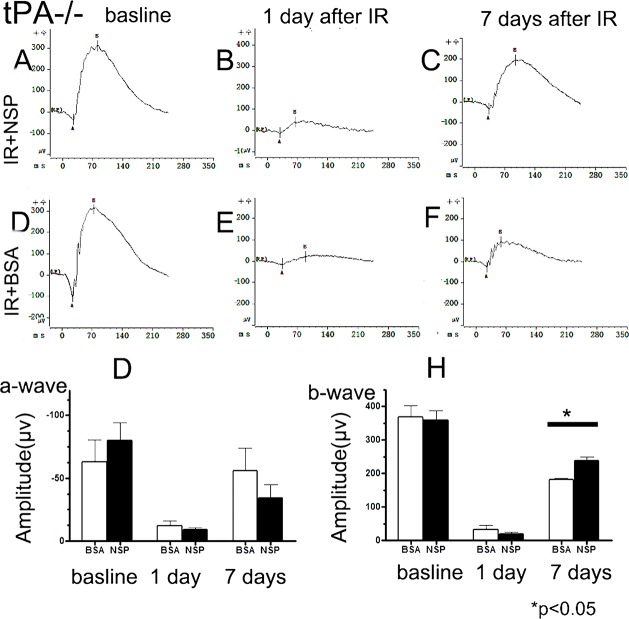
Effect of Neuroserpin on electroretinogram responses in tPA-/-mice. (A-C) Representative ERG of tPA-/- mice of the treatment assignment IR+NSP at baseline (before IR injury), 1 day and 7 days after injury, respectively. (D-F) Representative ERG of tPA-/- mice of the treatment assignment IR+BSA at baseline and 1 day and 7 days after ischemic retinal injury. Each ERG was obtained by averaging two responses to 2.5cd.s/m^2^ flashes with an interstimulus interval of 10 milliseconds; (G) Data analyses of ERG a-wave amplitudes for both treatment conditions at baseline and one day and seven days after retinal ischemic injury; (H) Data analyses of ERG b-wave amplitudes at baseline and one day and seven days after retinal ischemic injury. Data is expressed as a mean ±SE; n = 6; (*) *P*<0.05between responses in IR+BSA control and IR+NSP groups.

At baseline, scotopic ERG measurements performed on BSA and NSP-treated animals show similar a- and b-wave profiles. Twenty-four hours after IR injury, both treatment groups exhibited a-wave and b-wave decreases, with no between-group differences observed. Seven days post injury, a and b-wave amplitudes begin to rise in both groups though, once again, b wave restoration was greater in NSP pretreated animals than BSA-treated controls (*p* = 0.01) (Fig 3, S3 Table).At baseline, 24 hours and 7 days after IR injury, a-wave and b-wave amplitudes of the tPA-/- mice groups resembled wild type patterns(S3 Fig).

### IR injury-induced apoptosis is inhibited by NSP in tPA^-/-^mice

**Fig 4:**
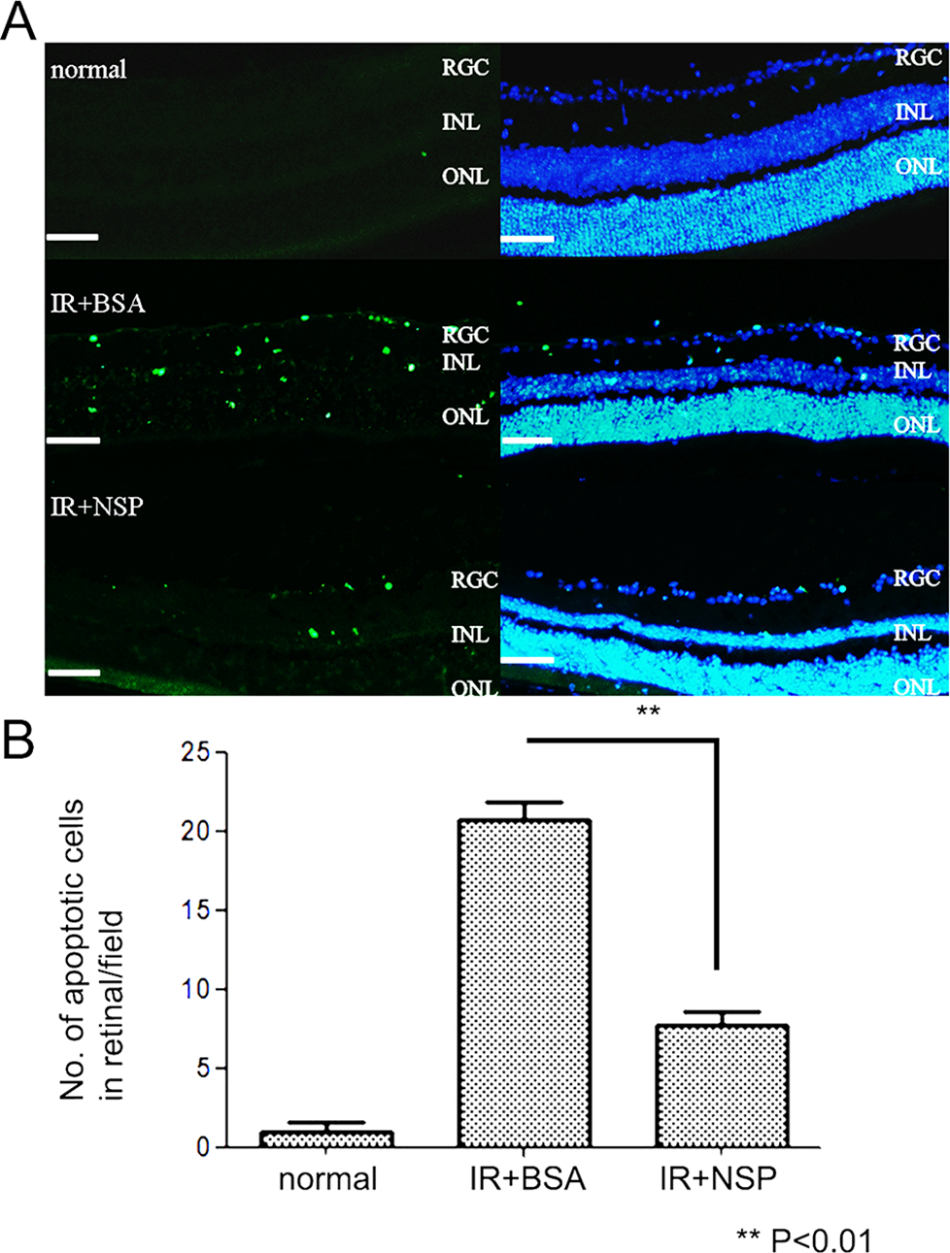
Apoptosis reduction in the retina 24 hours after IR injury in tPA^-/-^mice. (A) TUNEL analysis of retinas from three different treatment groups: normal control (top),IR+BSA control (middle) or IR+NSP group (bottom). Green indicates TUNEL-positive cells; blue indicates DAPI; RGC: retinal ganglion cell layer, INL: inner nuclear layer, ONL: outer nuclear layer. Scale bar, 50μm (B) Quantitative analysis of TUNEL-positive cells in retinas from different treatment groups. Data are presented as mean and error bars represent standard deviations (SD); Each group n = 3; Asterisks denote significant differences from the TUNEL levels of the BSA pretreated IR injury group and the NSP-treated injury control group; (**) *P*<0.01; NSP: neuroserpin, BSA: bovine serum albumin.

The pretreatment of NSP significantly reduced the number of TUNEL-positive nuclei in the retina 24 hours after IR injury as compared to the BSA-treated control group in tPA^-/-^mice. Quantitative analysis further confirmed a significant decrease in the number of TUNEL-positive nuclei in NSP pretreated groups (IR+NSP: 7.67±1.53 per field and IR+BSA control group: 20.67±2.08 per field; *p*<0.01; original magnification 100x) (Fig 4).Compared to tPA-/- mice, NSP pretreatment appeared to have a slightly higher protective effect in wild type mice, reducing more apoptosis, but the difference was not significant (S4 Fig).

### NSP-induced IR tolerance in the retina

**Fig 5:**
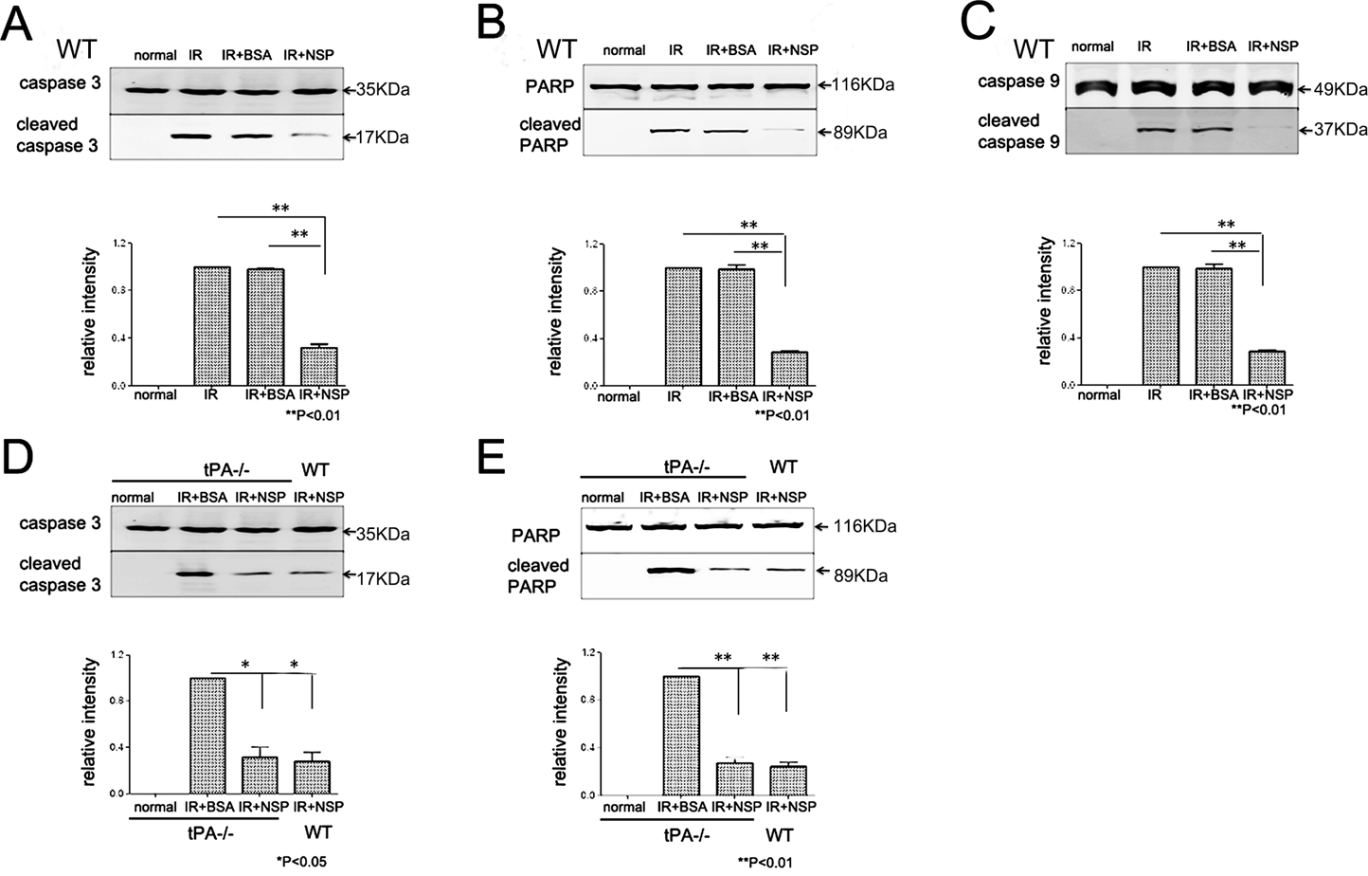
Expressions of caspase-3, PARP, caspase-9, and their cleaved forms 24 hours after IR injury. Western blot results of (A) caspase-3, cleaved caspase-3, and the relative expression of cleaved caspase-3 in wild type mice; (B) PARP, cleaved PARP, and the relative expression of cleaved PARP in wild type mice; (C)caspase-9,cleaved caspase-9, and the relative expression of cleaved caspase-9 in wild type mice. Significant differences in the relative intensity of cleaved caspase-3, cleaved PARP and cleaved caspase-9 were detected between NSP+IR group and BSA+IR and untreated IR. Results displayed as mean, error bars denote SD; n = 5 for each group; **P<0.01. Western blot results of (D) caspase-3, cleaved caspase-3, and the relative expression of cleaved caspase-3and (E) PARP, cleaved PARP, and relative cleaved PARP expression in tPA-/- mice. Significant difference were detected between the NSP+IR group and the BSA+IR group; NSP+IR levels of cleaved caspase-3 and cleaved PARP in tPA-/- were comparable to wild types.(**) *P*<0.01 (*) P<0.05 IR+NSP *vs*.IR+BSA group; NSP: Neuroserpin, WT: wild type mice, tPA-/-: tPA knockout mice

After IR injury, the cleaved forms of caspase-3, PARP and caspase-9 (all markers of apoptotic activation) were elevated in wild type mice. Pretreatment of animals with BSA did not appear to influence the expression of these apoptotic proteins in retinal tissue. Pretreatment with NSP, on the other hand, inhibited IR-induced elevations of these apoptotic proteins (cleaved caspase-3 by 65%, cleaved PARP by 70% and cleaved caspase-9 by 80%). Very similar trends were observed in the cleaved caspase-3and cleaved PARP detection of tPA-/- mice. In fact, NSP-pretreated tPA-/- animals attenuated the IR-induced elevation of cleaved caspase-3and cleaved PARP to the same effect as NSP-pretreated wild type mice (Fig 5). The levels of cleaved caspase-8, however, did not appear to improve with NSP-pretreatment after IR injury (S5 **Fig**).

### Endogenous NSP expression in the retina under normal and IR-induced pathology

**Fig 6:**
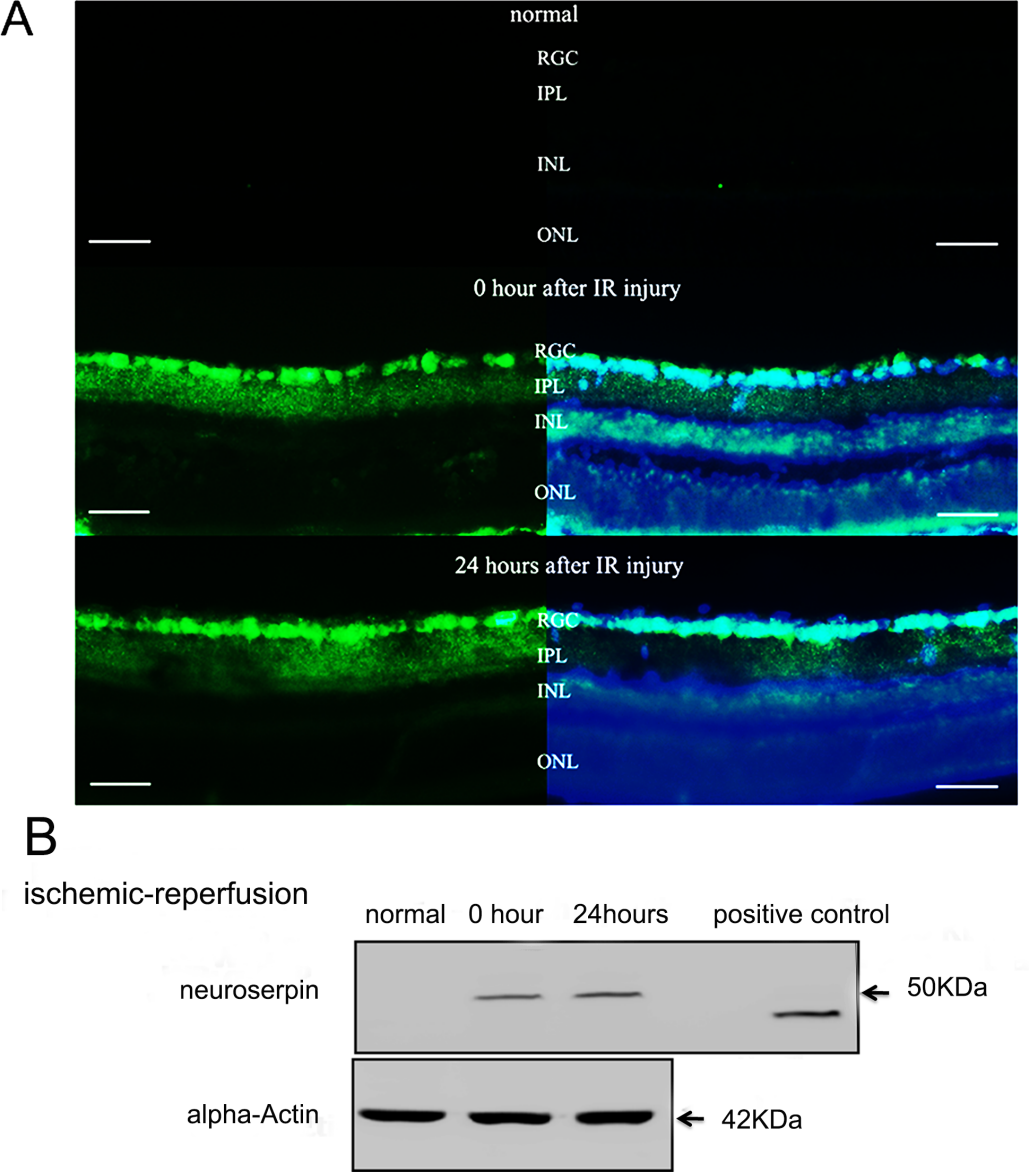
Endogneous NSP Expression in the retina (A) Immunofluorescent staining of retinal sections under normal (uninjured) conditions (top), immediately following IR injury (middle), and 24 hours following IR injury(bottom);neuroserpin immuneactivity (green) was detected by a FITC-labeled secondary antibody; cell nuclei (blue) were counter stained with DAPI; neuroserpin was mainly found at the retinal ganglion cell layer (RGC).(B) Western blot analysis showed that neuroserpin was barely detectable in normal uninjured retinas but increased expression at 0 and 24 hours following IR injury (human recombinant neuroserpin used as positive control, and alpha-actin as the loading control);NSP: neuroserpin, RGC: retinal ganglion cell layer, IPL: inner plexiform layer, INL: inner nuclear layer, ONL: outer nuclear layer. Scale bar, 50μm.

Immunofluorescence and Western blot were utilized to investigate the endogenous NSP expression profile of a healthy and IR-injured animal. NSP expression was virtually undetectable under normal physiological conditions but increased immediately following IR injury, and remained elevated 24 hours post-injury. The distribution of elevated NSP was mainly observed at the GCL (Fig 6).

## Discussion

In the present study, we discovered potential neuroprotective effect of NSP in the retina after IR injury. Given intravitreally, NSP can help preserve retinal function and combat the activation of apoptotic pathways and subsequent cell death. Wild type mice pre-treated with NSP specifically improved retinal function at a faster rate than controls. Further supporting the neuroprotective effect of intravitreal NSP via apoptotic resistance was the finding of reduced retinal cell apoptotsis unseen in BSA- pretreated injury groups. Also NSP reduced IR injury-induced activation of caspase-3 PARP and caspase-9 better than their control-treated counterparts. Interestingly, functional and apoptotic markers were improved by NSP pretreatment even in tPA null mice. Indicating that though neuroserpin is a biological inhibitor of tPA, this function might play little or no significant role in the neuroprotective action of NSP in IR induced retinal injury.

NSP, a member of the serpin (serine proteinase inhibitors) gene family, is reportedly found almost exclusively within the central nervous system [11,13,14,15,27]. It may interact with low density lipoprotein receptor-related protein (LRP) to help maintain the balance between proteases and inhibitors[28,29,30], regulate N-cadherin-mediated cell adhesion[30], inhibit vascular permeability[15,31,32,33], reduce cerebral infarct volume and protect neurons from ischemia-induced apoptosis[16,17,34]. As a natural inhibitor of tPA, NSP is believed to work through the inhibition of tPA activity. And the human recombinant neuroserpin used in our present research demonstrated the ability to inhibit tPA's proteolytic activity in vitro (S6 Fig).Gelderblom Mdescribed that in an animal model of temporal focal ischemic stroke, infarct size and neurological outcome were worse in neuroserpin deficient mice because of the increasing activity of tPA and excessive microglial activation[35].Also Ma et al have found that neuroserpin protected neurons against oxygen-glucose deprivation and reoxygenation mainly by inhibiting tPA-mediated acute neuronal excitotoxicity[36].On the other hand, Lee TW et al reported that neuroserpin mutants lacking tPA inhibitory activity maintained the ability to regulate the levels of N-cadherin available for construction, maintenance and control of synapses and synaptic dynamics in nerve cells, independent of tPA inhibitor activity[37]. This suggests that neuroserpin may also function through channels beyond the inhibition of tPA. Furthermore, Wu J et al found that the neuroprotective effect of neuroserpin might be due to inhibition of plasmin-mediated excitotoxin-induced cell death and therefore independent from its inhibitory tPA activity. This suggests that the neuroprotective effect of NSP may occur through other pathways beyond tPA inhibition [23]. However, there is a scarcity of literature in the area of the neuro-protective capacity of NSP in the retina in general.

Results from this study demonstrate that ischemic retinal injury, as determined by morphometric and ERG analysis, is significantly reduced by1 μL NSP solution (20 μmol/L).After IR-injury, apoptosis was detected at all retinal layers. Intravitreal NSP greatly reduced the number of apoptotic cells, this was consistent with the previous findings of Yepes et al[16], which showed that neuroserpin administration decreased the number of apoptotic cells in the ischemic penumbra. Additionally, the observed reduction in apoptosis was accompanied by a restoration of retinal b-waves. This preference may be the result of the intravitreal administration method used to deliver NSP, which would theoretically skew in the direction of the inner retina, thus affecting the bipolar cells (which generate b-waves) of the inner nuclear layer more, and the photoreceptor (which generate a-waves)less.

A significant decrease of activated caspase-3 and its main moderator-caspase 9 was also found in this study. As previously reported, Caspase-3, a pivotal driver of apoptosis, plays a critical role in retinal degeneration including ischemia and light injury[38,39].Caspase-9 is found to play an important role in retinal ganglion cell death in axotomized injury[40]and glaucoma[41].It also acts as the main pathway of retinal capillary cell apoptosis in diabetic retinas[42]and in neuron death occurring from serum deprivation[43].In our study, caspase-3, PARP (the substrate of caspase-3) and caspase-9 (the modulator of the caspase-3) were all significantly up-regulated after retinal IR injury, which was consistent with previous findings in liver and brain IR injury models[44,45].Previous studies have shown that neuroserpin administration decreased the number of apoptotic cells in the ischemic penumbra[16]. Here, caspase-3, casapse-9 and PARP, when increased by IR injury, were significantly reduced by NSP in wild type and tPA-/- mice. The reduction was accompanied by a reduction in apoptosis. This suggests that NSP might protect retina from IR injury through the inhibition of caspase-3 activation via the caspase-9 cell death signal pathway.

To assess whether NSP’s neuroprotection was mediated by its action as a tPA inhibitor, tPA-/- mice were studied alongside wild type. No differences were found between wild type and tPA-/- mice in their functional or apoptotic responses to NSP treatment in the context of IR retinal injury. The results further suggest that NSP exerts a neuroprotective effect in the retina in the face of IR injury. Moreover, this protective role appears independent of tPA inhibition. This observation does not rule out that endogenous inhibition of tPA by NSP may also contribute to neural protection in other ischemic injury models, as has been reported. Though not statistically different, our results suggest that the effect of NSP in tPA-/- mice is slightly less favorable than that of wild type. This would indicate that without tPA, the neuroprotective effect of NSP maybe reduced, though more conclusive evidence is required. Altogether, the neuroprotective effect of NSP might be more diverse than previously thought, working not only through the inhibition of tPA but through other pathways as well. However by comparing with previous researches, we found that recombinant neuroserpin which was not expressed in E.Coli demonstrated a relatively higher efficiency of inhibiting tPA than the one we used this time, which was expressed in E.Coli. This might be related to the misfolding due expression in E. Coli[11,46,47].

The pathological changes that occur in the retina after IR injury share a high degree of similarity with those of stroke in the central nerve system [48,49]. The interruption of blood supply to the brain and retina can result in a wide variety of metabolic derangements including: elevation of Ca^2+^ levels, excitotoxicity, energy failure, generation of free radicals, blood barrier breakage, inflammation and neuronal apoptosis [50,51,52]. In the present study, the actions of NSP pretreatment were similar to those previously reported in injury models of stroke[17,24,25]. Also as indicated by immunofluorescence and Western blot analysis, endogenous NSP expression increases significantly after IR injury as that in the models of stroke[16,24]. This suggests that NSP might act as a natural protective element against IR injury under pathological conditions.

## Conclusions

The work reported herein supports previous evidence regarding the neuroprotective potential of NSP in ischemic injury. Given intravitreally, NSP can increase the tolerance of the retina and reduce the number of apoptotic neurons after IR injury. This action was observed in wild type as well as tPA-/- mice indicating that the action of NSP is independent of tPA inhibition and might instead act (at least partially) through the inhibition of the caspase-9 cell death regulatory pathway. Though plausible, a direct link between NSP and the inhibition of caspase-3 remained to be investigated. Further study may provide more information about the mechanisms which mediate NSP neuroprotection. As a natural substance expressed in the retina under pathological conditions, NSP supplementation may prove to have promising therapeutic value in the treatment of retinal diseases, especially those characterized by ischemia or apoptosis.

## Supporting Information

S1 Fig Western blot for tPA in retinal from wild type and tPA-/-mice. The expression of tPA in retina from tPA-/- mice was tested and wild type mice in normal, IR, IR+BSA and IR+NSP groups were served as control. tPA was not found in tPA-/- mice, but present in wild type groups. IR: ischemic-reperfusion, NSP: neuroserpin, BSA: bovine serum albumin.
(TIF)

S2 Fig Western blot for level of endogenous neuroserpin in retina after IR injury. Retinal homogenate (1μl) from 0 and 24 hours after ischemic-reperfusion injury were used for western blot, and 0.1μM human recombinant neuroserpin at different volumes (2μl,6μl and 12μl) were used as control. Gray value ratio was calculated. The level of endogenous neuroserpin was about 0.38–0.42x10-4 μmol/g, which could be equivalent to 0.38–0.42μM assuming a tissue density of 1 g/ml.
(TIF)

S3 Fig The effect of NSP on ERG responses in tPA-/- and wildtype mice. Wide type and tPA-/- mice demonstrated similar ERG a-wave (left) and b-wave(right) amplitudes at baseline, one day and seven days after NSP pretreatment and retinal ischemic injury. Data is expressed as a mean +SE; n = 6,NSP: neuroserpin.
(TIF)

S4 Fig NSP demonstrated similar neuroprotective ability in tPA-/- and wild type mice. 24 hours after IR injury, TUNEL-positive cells was found in retinas, pretreatment of NSP greatly decreased the number of TUNEL-positive cells, wide type and tPA-/- groups show similar results. Data presented as mean and error bars represent standard deviations (SD); each group n = 3.IR: ischemic-reperfusion, NSP: neuroserpin, BSA: bovine serum albumin.
(TIF)

S5 Fig Expressions of caspase-8 and cleaved caspase-8 following IR injury. There was no difference in expression of capase-8 in all groups, and ischemic-reperfusion injury didn’t induced caspase-8 cleavage both in wide type and tPA-/- mice. NSP: neuroserpin, BSA: bovine serum albumin.
(TIF)

S6 Fig Human recombinant neuroserpin can inhibit tPA's proteolytic activity. (A) tPA dissolving fibrinplate in a dose-depend way. tPAin higher concentration could dissolve larger circle of fibrin plate, the lowest concentration of tPA dissolving fibrin plate was 3.906ng/ul.(B) neuroserpin inhibiting tPA' s ability of dissolving fibrin plate. 25ug human recombinant neuroserpin reduced 3.90625ng/ultPA(10ul) from dissolving fibrin plate (with 10ul 3.90625ng/ul tPA solutions alone as control).
(TIF)

S1 Table The number of animals used in present study. (DOC)

S2 Table Effect of neuroserpin on electroretinogram responses in wild type mice (2.5cd.s/m2 flashes with an interstimulus interval of 10 milliseconds). (DOCX)

S3 Table Effect of neuroserpin on electroretinogram responses in tPA-/- mice (2.5cd.s/m2 flashes with an interstimulus interval of 10 milliseconds) (DOC)
