# Supplementary figures and images for: Retina Is Protected by Neuroserpin from Ischemic/Reperfusion-Induced Injury Independent of Tissue-Type Plasminogen Activator
Source: PLoS One. 2015 Jul 15;10(7):e0130440. doi: 10.1371/journal.pone.0130440 (PMC4503687; doi:10.1371/journal.pone.0130440)

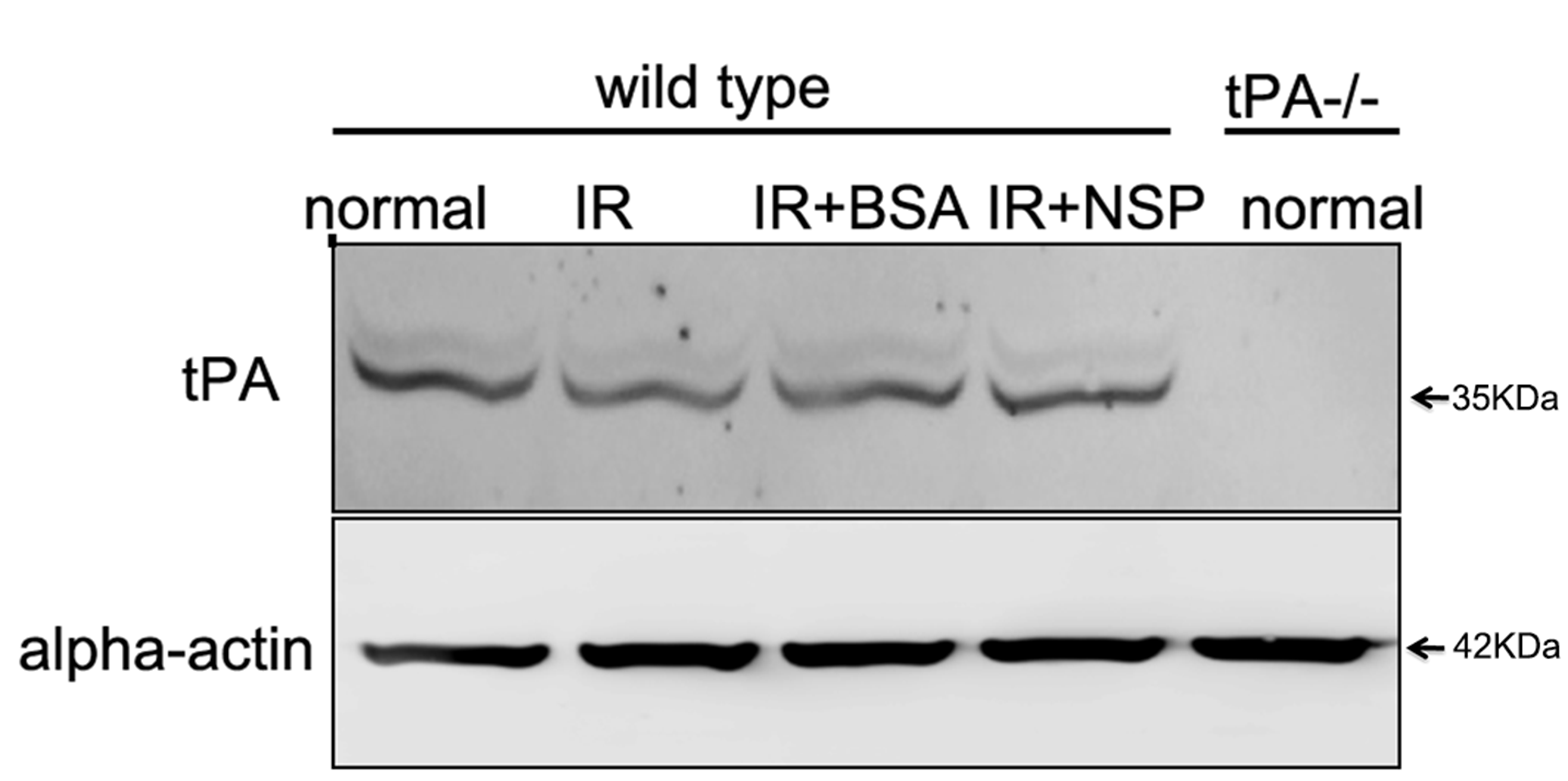

Supplement: S1 Fig — The expression of tPA in retina from tPA-/- mice was tested and wild type mice in normal, IR, IR+BSA and IR+NSP groups were served as control. tPA was not found in tPA-/- mice, but present in wild type groups. IR: ischemic-reperfusion, NSP: neuroserpin, BSA: bovine serum albumin. (TIF) [file pone.0130440.s001.tif]

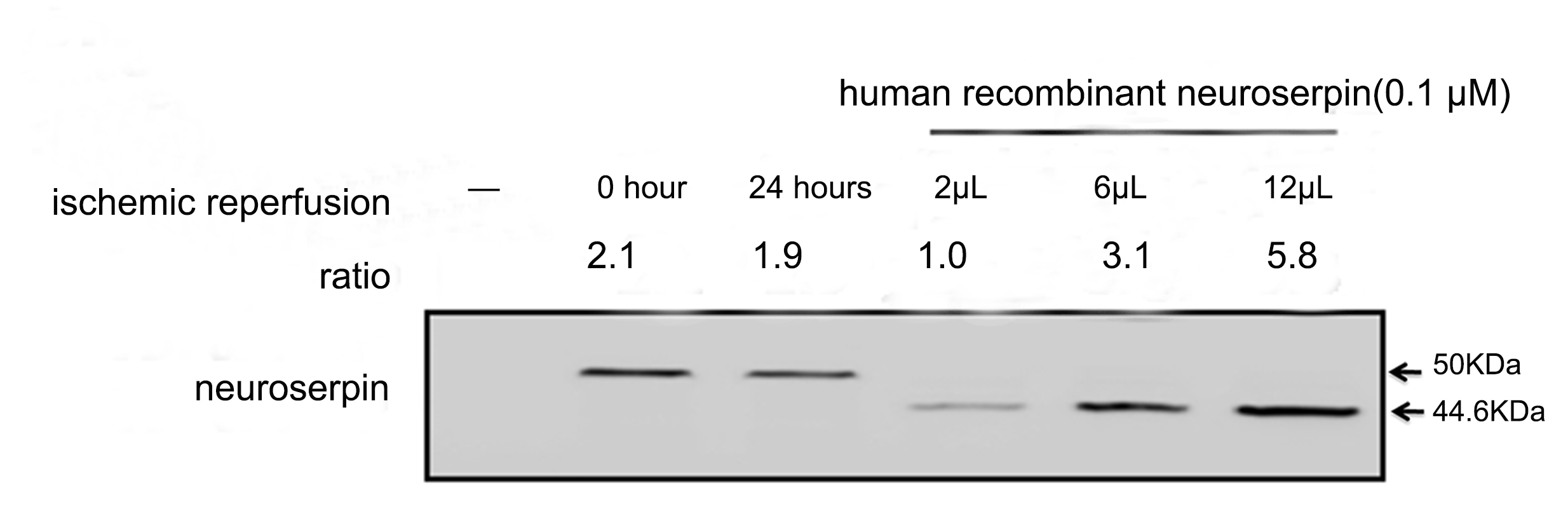

Supplement: S2 Fig — Retinal homogenate (1μl) from 0 and 24 hours after ischemic-reperfusion injury were used for western blot, and 0.1μM human recombinant neuroserpin at different volumes (2μl,6μl and 12μl) were used as control. Gray value ratio was calculated. The level of endogenous neuroserpin was about 0.38–0.42x10-4 μmol/g, which could be equivalent to 0.38–0.42μM assuming a tissue density of 1 g/ml. (TIF) [file pone.0130440.s002.tif]

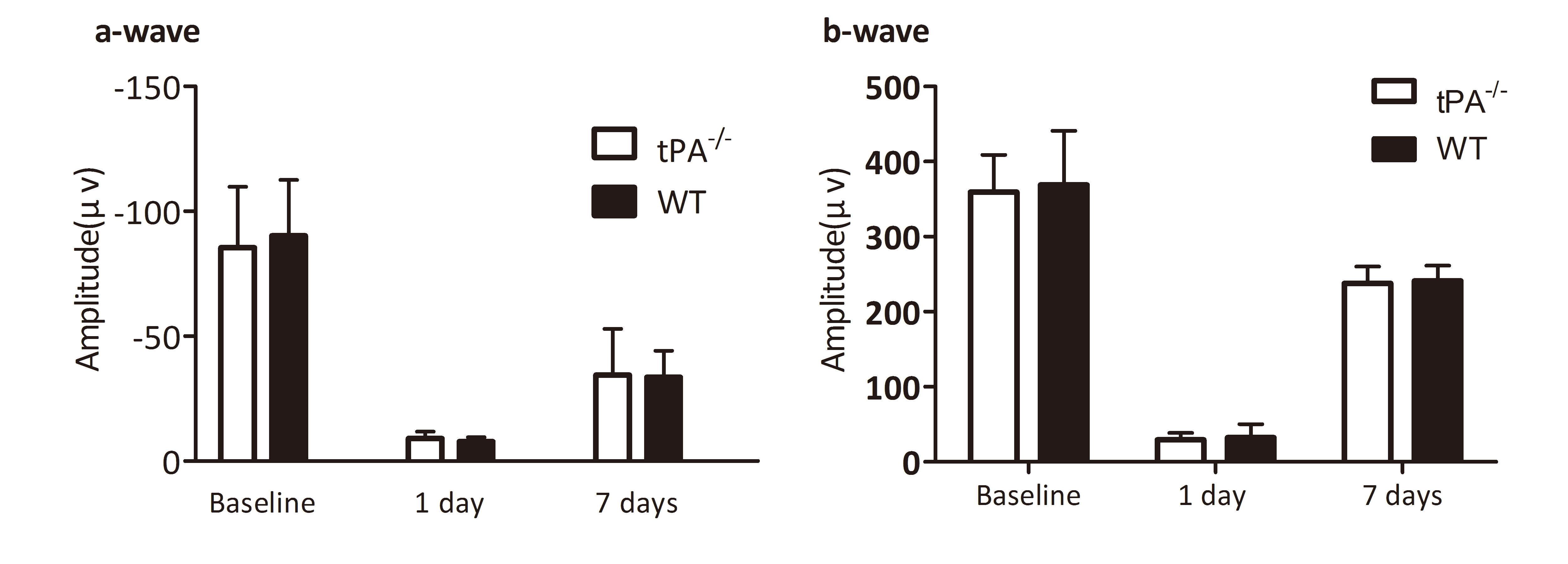

Supplement: S3 Fig — Wide type and tPA-/- mice demonstrated similar ERG a-wave (left) and b-wave(right) amplitudes at baseline, one day and seven days after NSP pretreatment and retinal ischemic injury. Data is expressed as a mean +SE; n = 6,NSP: neuroserpin. (TIF) [file pone.0130440.s003.tif]

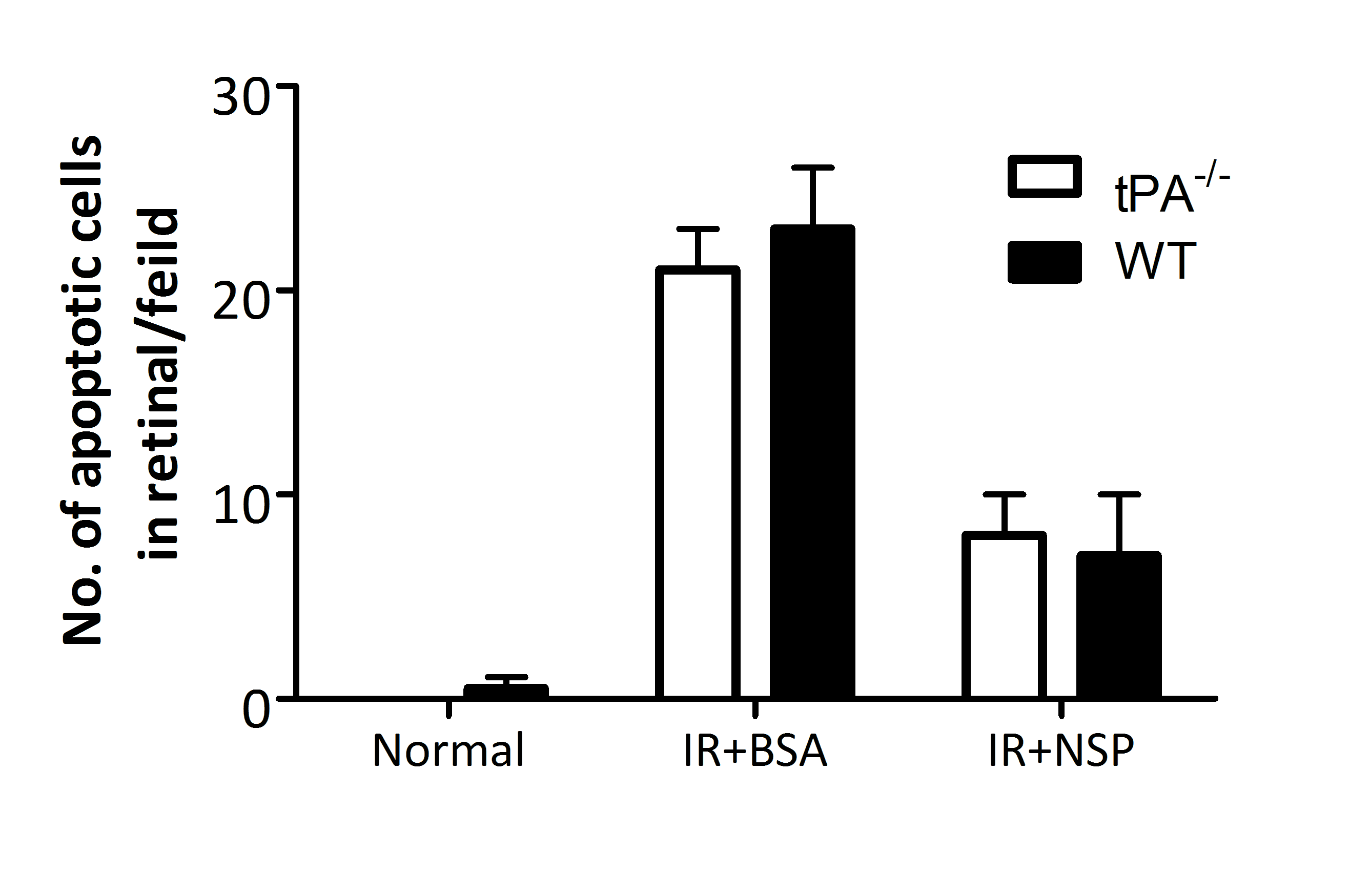

Supplement: S4 Fig — 24 hours after IR injury, TUNEL-positive cells was found in retinas, pretreatment of NSP greatly decreased the number of TUNEL-positive cells, wide type and tPA-/- groups show similar results. Data presented as mean and error bars represent standard deviations (SD); each group n = 3.IR: ischemic-reperfusion, NSP: neuroserpin, BSA: bovine serum albumin. (TIF) [file pone.0130440.s004.tif]

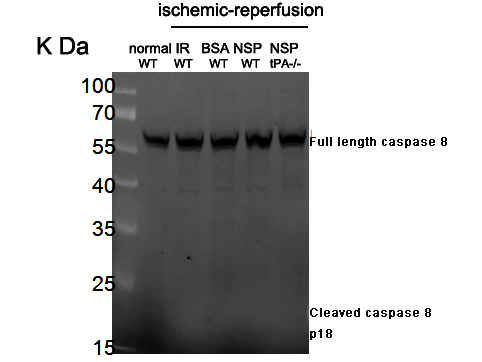

Supplement: S5 Fig — There was no difference in expression of capase-8 in all groups, and ischemic-reperfusion injury didn’t induced caspase-8 cleavage both in wide type and tPA-/- mice. NSP: neuroserpin, BSA: bovine serum albumin. (TIF) [file pone.0130440.s005.tif]

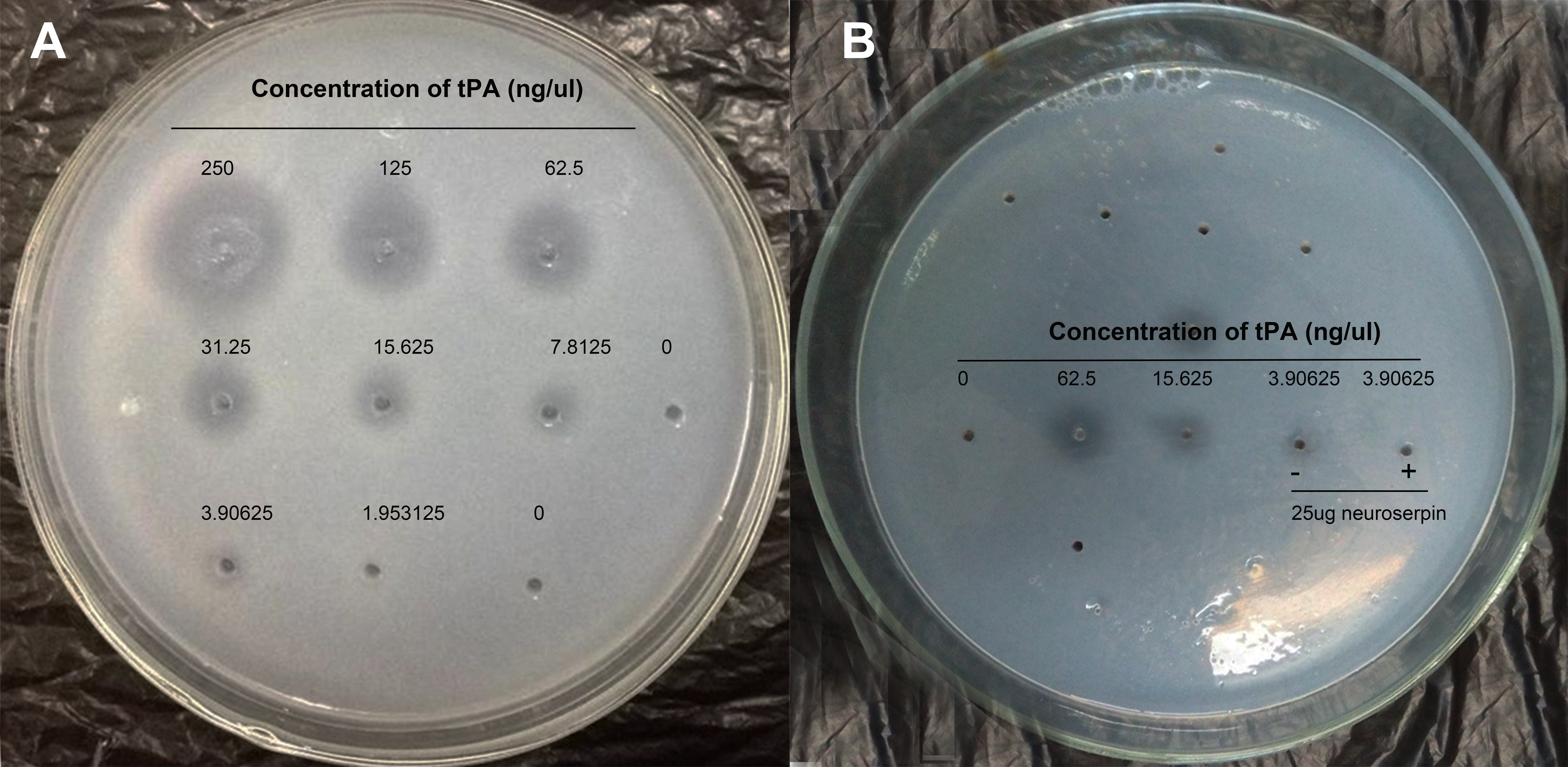

Supplement: S6 Fig — (A) tPA dissolving fibrinplate in a dose-depend way. tPAin higher concentration could dissolve larger circle of fibrin plate, the lowest concentration of tPA dissolving fibrin plate was 3.906ng/ul.(B) neuroserpin inhibiting tPA' s ability of dissolving fibrin plate. 25ug human recombinant neuroserpin reduced 3.90625ng/ultPA(10ul) from dissolving fibrin plate (with 10ul 3.90625ng/ul tPA solutions alone as control). (TIF) [file pone.0130440.s006.tif]
