# Supplementary material for: Retina Is Protected by Neuroserpin from Ischemic/Reperfusion-Induced Injury Independent of Tissue-Type Plasminogen Activator
Source: PLoS One. 2015 Jul 15;10(7):e0130440. doi: 10.1371/journal.pone.0130440 (PMC4503687; doi:10.1371/journal.pone.0130440)
Supplement: S1 Table — (DOC) [file pone.0130440.s007.doc]

**S1 Table . The number of animals used in present study**

| **The number of animals used in present study** | |  |
| --- | --- | --- |
| animals | process | numbers(n) |
| wide type | ERG | 12 |
|  | TUNEL | 12 |
|  | western blot for apoptotic signal pathway | 20 |
|  | NSP expression through immunofluorescence | 9 |
|  | NSP expression through western blot | 9 |
| tPA-/- mice | ERG | 12 |
|  | TUNEL | 9 |
|  | western blot for apoptotic signal pathway | 15 |
