# Supplementary material for: Retina Is Protected by Neuroserpin from Ischemic/Reperfusion-Induced Injury Independent of Tissue-Type Plasminogen Activator
Source: PLoS One. 2015 Jul 15;10(7):e0130440. doi: 10.1371/journal.pone.0130440 (PMC4503687; doi:10.1371/journal.pone.0130440)
Supplement: S2 Table — (DOCX) [file pone.0130440.s008.docx]

**S2 Table . Effect of Neuroserpin on electroretinogram responses in wild type mice (**2.5cd.s/m^2^ flashes with an interstimulus interval of 10 milliseconds**).**

| **WT mice** | **waves** | **Baseline (μv)** | **NSP *vs.* BSA** | **1 days after IR(μv)** | **NSP *vs.* BSA** | **7 days after IR(μv)** | **NSP *vs.* BSA** |
| --- | --- | --- | --- | --- | --- | --- | --- |
| BSA-treated | a-wave | -89.77+31.28 | p= 0.76 | -5.091+2.25 | p= 0.20 | -29.6+2.41 | P=0.58 |
| NSP-treated |  | -90.05+22.43 |  | -7.65+1.82 |  | -33.40+10.71 |  |
| BSA-treated | b-wave | 357.97+60.45 | p=0.85 | 36.96+23.56 | p=0.80 | 158.67+34.82 | P=0.02 |
| NSP-treated |  | 369.13+71.82 |  | 32.33+17.81 |  | 241.17+20.17 |  |
