# Supplementary material for: Retina Is Protected by Neuroserpin from Ischemic/Reperfusion-Induced Injury Independent of Tissue-Type Plasminogen Activator
Source: PLoS One. 2015 Jul 15;10(7):e0130440. doi: 10.1371/journal.pone.0130440 (PMC4503687; doi:10.1371/journal.pone.0130440)
Supplement: S3 Table — (DOC) [file pone.0130440.s009.doc]

**S3 Table . Effect of Neuroserpin on electroretinogram responses in tPA-/- mice (2.5cd.s/m^2^ flashes with an interstimulus interval of 10 milliseconds).**

| **tPA-/-mice** | **waves** | **Baseline(μv)** | **NSP *vs.* BSA** | **1 days after IR(μv)** | **NSP *vs.* BSA** | **7 days after IR(μv)** | **NSP *vs .*BSA** |
| --- | --- | --- | --- | --- | --- | --- | --- |
| BSA-treated | a-wave | -63.21+29.8 | p= 0.48 | -12.22+6.64 | p= 0.48 | -59.08+30.83 | P=0.35 |
| NSP-treated |  | -85.33+24.31 |  | -8.91+2.84 |  | -34.31+18.50 |  |
| BSA-treated | b-wave | 368.1+60.41 | p=0.86 | 32.24+24.02 | p=0.44 | 181.5+7.62 | P=0.01 |
| NSP-treated |  | 359.47+49.32 |  | 29.49+8.87 |  | 237.67+22.41 |  |
